# Trapping and Propelling Microparticles at Long Range by Using an Entirely Stripped and Slightly Tapered No-Core Optical Fiber

**DOI:** 10.3390/s130302884

**Published:** 2013-02-28

**Authors:** Fang-Wen Sheu, Yen-Si Huang

**Affiliations:** Department of Electrophysics, National Chiayi University, Chiayi 60004, Taiwan; E-Mail: s0990210@mail.ncyu.edu.tw

**Keywords:** fiber optics, evanescent wave, optical manipulation

## Abstract

A stripped no-core optical fiber with a 125 μm diameter was transformed into a symmetric and unbroken optical fiber that tapers slightly to a 45-μm-diameter waist. The laser light can be easily launched into the no-core optical fiber. The enhanced evanescent wave of the slightly tapered no-core optical fiber can attract nearby 5-μm-diameter polystyrene microparticles onto the surface of the tapered multimode optical fiber within fast flowing fluid and propel the trapped particles in the direction of the light propagation to longer delivery range than is possible using a slightly tapered telecom single-mode optical fiber.

## 1. Introduction

With the conventional optical tweezers technique [1], we can trap microparticles stably in three dimensions by producing a strongly focused laser beam. Alternatively, the evanescent wave from an integrated optical waveguide can also be used to attract and transport microparticles [2–6], especially for a submicrometer waveguide with high-intensity evanescent field and enhanced optical propelling effect on microparticles. Nevertheless, the fabrication process of a submicrometer strip waveguide is tedious. Similarly, the evanescent wave optical trapping and propulsion of microspheres along subwavelength ultra-thin optical fibers has also been demonstrated [7–12]. In this approach, a short section of the single-mode optical fiber was tapered to submicrometer dimensions such that a significant fraction of the propagating mode coupled from the core to the cladding can penetrate an appreciable distance into the surrounding medium around the surface of the highly tapered optical fiber and thus can be exploited for the optical trapping and propelling of individual and clustered microparticles. Microparticles around the tapered nanofiber surface can be trapped by the optical gradient force of the evanescent filed and propelled by the scattering force in the same direction as the laser light propagates [12]. The tapered fiber has the benefit of higher configuration flexibility and lower optical insertion loss compared with the planar integrated waveguide. Nevertheless, a heavily tapered optical fiber (a subwavelength nanofiber) needs very careful preparation and has many difficulties during the manipulation process. Therefore, we have previously proposed and demonstrated a technique for attracting and transporting microparticles using the evanescent wave of a slightly tapered telecom single-mode optical fiber with a 50 μm diameter at the minimum waist, which is easily manufactured [13].

Here, we report the optical attraction and propulsion of microparticles using the evanescent wave of a slightly tapered no-core optical fiber, which behaves as a tapered multimode optical fiber and has some additional benefits. In comparison with a telecom single-mode optical fiber [13], it is much easier to couple the laser light into a no-core optical fiber. Further, since the glass rod that constitutes a no-core optical fiber is an optical waveguide that has an evanescent wave along its entire surface, using a slightly tapered no-core optical fiber for trapping and propelling microparticles results in longer delivery range. On the other hand, in comparison with a heavily tapered optical fiber (subwavelength optical wire) [7–12], because the diameter of the slightly tapered no-core optical fiber is much larger than the optical wavelength of the coherent laser light, the numerous high-order modes in the multimode no-core optical fiber will result in many tiny interfering optical speckles and provide a more uniform evanescent wave around the fiber surface, which may allow microparticles to be attracted and propelled with forces that have a smoother spatial distribution. As a result, the few-mode interference beating effect [14] can be avoided in performing the evanescent wave trapping and propelling of microparticles by a slightly tapered no-core optical fiber.

## 2. Experimental System and Methods

We made use of a fiber puller (P-2000, Sutter Instrument Co., heating source: CO_2_ Laser, Novato, California, CA, USA) to transform a no-core optical fiber (POFC NCF125, Prime Optical Fiber Corp., Miao-Li, Taiwan) into a symmetric and unbroken slightly tapered optical fiber. The diameter of the original no-core optical fiber is ∼125 μm and the waist diameter of the tapered no-core optical fiber is measured to be ∼45 μm by using an extra optical microscope. The no-core optical fiber is made of silica glass just like the telecom single-mode optical fiber [13], but it has a homogeneous refractive index distribution (that is, it is a tiny pure silica glass rod without doping).

**Figure 1.**
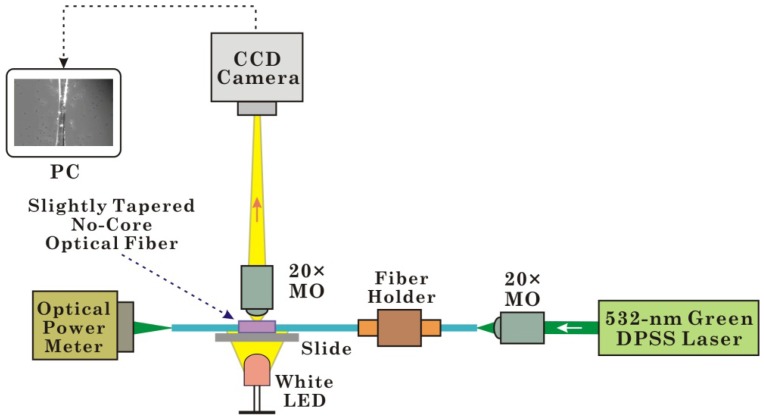
The configuration of the experimental setup for trapping and propelling microparticles with the evanescent field of a slightly tapered no-core optical fiber. MO, microscope objective lens; PC, personal computer; CCD, charge-coupled device; DPSS, diode-pumped solid-state; and LED, light-emitting diode.

The experimental setup for trapping and propelling microparticles with a slightly tapered no-core optical fiber is shown in Figure 1. The front end of the optical fiber (the untapered region) is fixed with a fiber holder, and the rear end is directed toward an optical power meter. The fiber taper region is positioned horizontally in a microfluidic channel that is formed by two pieces of adhesive tape, on a microscope slide. Light from a 532-nm green diode-pumped solid-state (DPSS) laser (GLM-L3IF-100, Unice E-O Services Inc., Taoyuan, Taiwan) is coupled into the slightly tapered no-core optical fiber through a 20× microscope objective (MO) lens. Then we use another 20× MO and a CCD camera (WAT-902H2, Watec Co., Tsuruoka, Japan) to observe the optical microscopic image of the slightly tapered no-core optical fiber from the top. The majority of the illumination is from the white light LED, which shines up through the slide from beneath.

**Figure 2.**
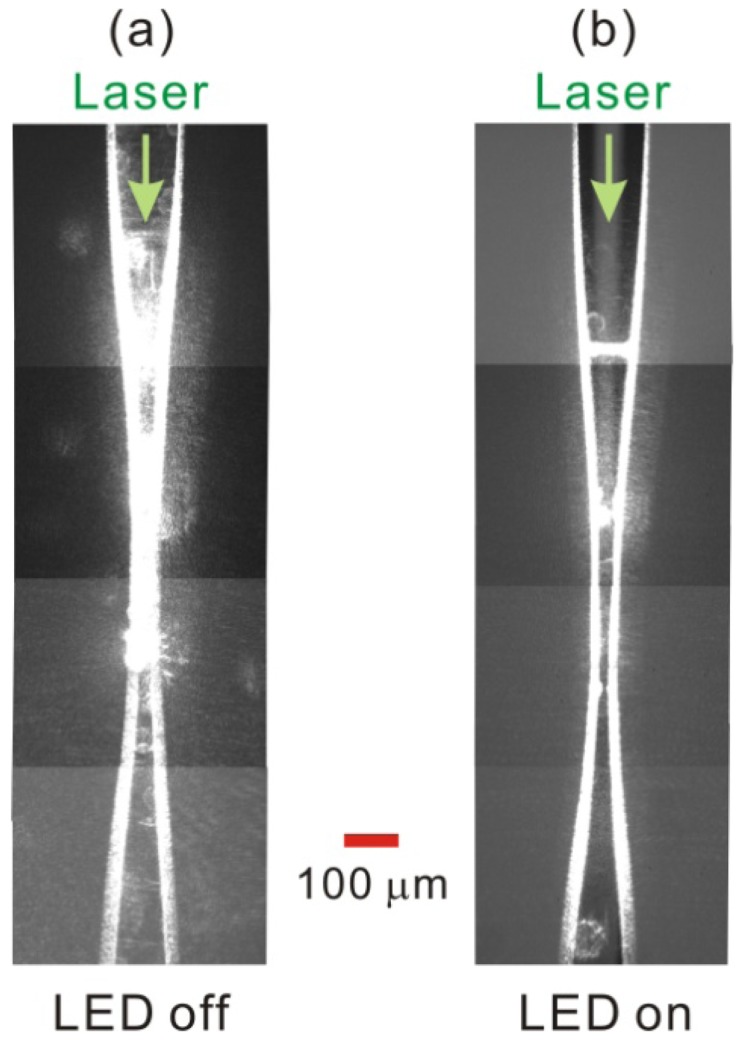
The observed optical microscopic images of the slightly tapered no-core optical fiber when the illumination of the LED is (**a**) turned off and (**b**) turned on, respectively.

Before being immersed in the solution of polystyrene microparticles, the slightly tapered no-core optical fiber that was coupled to the green laser light was first observed through our homemade microscope imaging system. Figure 2 shows the observed optical microscopic images of the slightly tapered no-core optical fiber when the LED illumination is turned off and turned on, respectively. To allow the observation of longer-range transportation of microparticles, we utilized a 20× MO as the imaging lens instead of a 50× long-working-distance MO [13]. Nevertheless, to avoid obtaining an image in which the microparticles appear to be too small, we did not utilize a 10× MO as the imaging lens. Therefore, the two superposed pictures in Figure 2 are cascades of four snapshot images captured separately at different fiber positions, resulting in a combined image that spans a fiber length of ∼1,350 μm. Figure 2(a) shows that, in the absence of LED illumination, the scattered light (green laser) of the slightly tapered no-core optical fiber is very strong at the front half of the tapered region of the fiber, which may hinder us from clearly observing the microparticles trapped there. The scattered light comes from some residual polymer coating defects and inhomogeneous smoothness on the tapered fiber surface [13]. Figure 2(b) shows that the background illumination of the white LED light can be bright enough to overwhelm the green laser light (with CCD automatic gain control) such that we can observe more clearly the trapped microparticles around the fiber taper section. On the other hand, Figure 2(a) also shows that the evanescent waves in the rear half of the fiber taper and in the two untapered sections of the no-core optical fiber are slightly weaker than those in the front half of the fiber taper, but they are still relatively strong and have an intensity scale capable of trapping and propelling microparticles, which will be demonstrated in the following experimental results.

Then we prepared the sample to perform the experiments on microparticle manipulation. The polystyrene microparticles with diameters of 5 μm were diluted in de-ionized water, and several drops of the suspension were placed with a pipette on the fiber taper section, which was positioned horizontally in the microfluidic channel on the microscope slide. Through the homemade microscope imaging system with the LED background illumination, we observed that, due to the evanescent wave around the slightly tapered no-core optical fiber, the microparticles were attracted and trapped on the fiber surface when the microparticles flowed near the slightly tapered no-core optical fiber, and then the microparticles were propelled by the radiation pressure, causing them to move along the slightly tapered no-core fiber in the direction of the light propagation. Because the viscous drag force of the surrounding water suspension counteracts the optical propulsion force, the microparticles can be accelerated only up to a constant terminal speed, where the forces exactly balance.

## 3. Experimental Results

**Figure 3.**
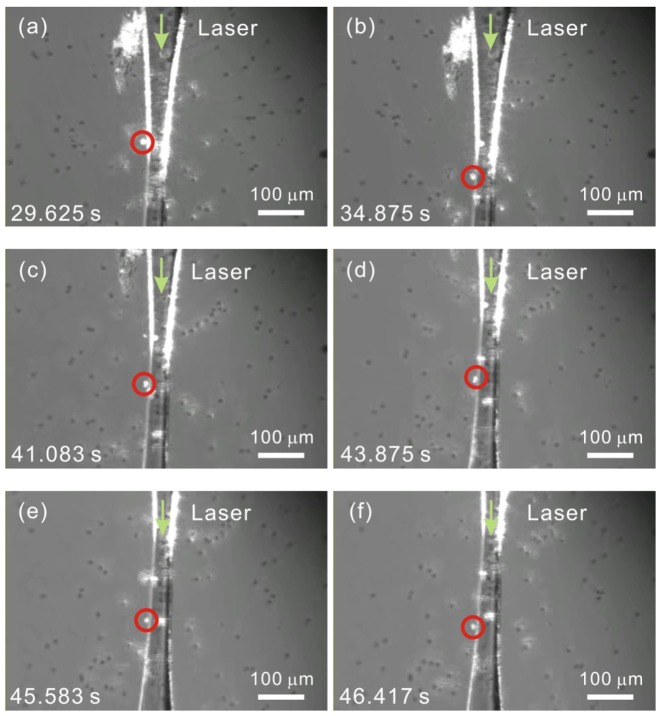
The observed microscopic images (derived from Media 1) of the transportation of a trapped microparticle (marked with a red circle) along the surface of the slightly tapered no-core optical fiber, which was positioned horizontally in the microfluidic channel on the microscope slide. The frame time in the movie is marked on the left-down corner of each image. Because the trapped microparticle was propelled at long range, the recorded images were taken at several different regions of the tapered fiber. Some notes about the particle transportation information are also added onto the recorded video.

Based on the aforementioned configuration, we observed the phenomena of the optical attraction and transportation of polystyrene microparticles along the surface of the slightly tapered no-core optical fiber in the direction of the light propagation. The 532-nm green laser light with an optical power of 90 mW was launched into the input end of the slightly tapered no-core optical fiber, and the fiber output power was measured to be 45 mW. In addition to the coupling loss at the input end of the fiber, the other optical loss originated mainly from the scattered light around the surface of the tapered no-core optical fiber, especially around the front fiber taper. Though the profile of the slightly tapered no-core optical fiber (maximum taper angle ∼5.5°) is in the lossy regime defined for a tapered nanofiber [14], yet the fiber waveguide structures and sizes are different and the total optical loss of the tapered fiber is merely ∼3 dB, hence the optical loss due to the leaky mode wave doesn't seem to be very high. In order to obtain the enhanced evanescent wave, the slightly tapered no-core optical fiber was not optimized thus some leaking light from the tapered fiber was spread out causing the scattering of the microparticles that were ∼50–100 μm away from the tapered fiber, which will be shown in the following experimental results. No optical filter was used in front of the CCD camera, and the white LED illumination was turned on, so we could observe clearly both the scattered light and the trapped microparticles around the surface of the slightly tapered no-core optical fiber, as shown in Figure 3, which is derived from the captured movie (Media 1). The frame time in the movie is marked on the left-down corner of each image (from time 29.625 s to 46.417 s). The microscopic images in Figure 3(a,b) shows that, within an interval of 5.25 s (from time 29.625 s to 34.875 s), the highly shining trapped microparticle marked with a red circle moved a distance of ∼91.78 μm along the surface of the slightly tapered no-core optical fiber without escaping. The average propulsion velocity of the microparticle over this range was thus calculated to be 17.48 μm·s^−1^ along the light propagation direction.

**Figure 4.**
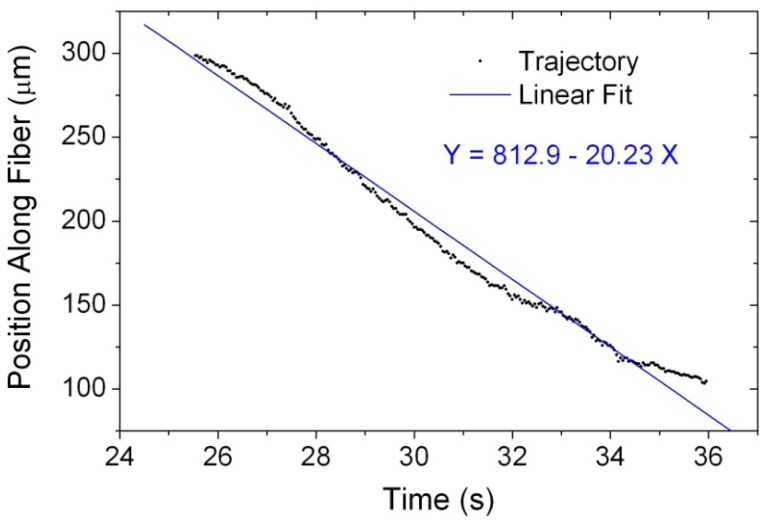
The temporal trajectory of the trapped and propelled microparticle along the slightly tapered no-core optical fiber *versus* the recording time from 25.542 s to 35.958 s, which is derived from the video analysis result using the particle-tracking software.

Alternatively, we have also utilized the video-based particle-tracking software “Tracker” [15] to perform the quantitative particle video analysis [12]. Figure 4 shows the temporal trajectory of the trapped and propelled microparticle along the slightly tapered no-core optical fiber *versus* the recording time from 25.542 s to 35.958 s. Because the trapped microparticle was propelled at long range, the recorded images were taken at several different regions of the tapered fiber. After the time 36.0 s, the fiber position sampled by the CCD camera was moved successively during the video recording process, prohibiting us from performing the particle video analysis any more. From the particle trajectory analysis result in Figure 4, we can find that the trapped microparticle moved at a somewhat uniform velocity and the averaged propulsion velocity was calculated to be approximately 20.23 μm·s^−1^ from a linear fit to the trajectory, with a similar scale like that reported in Reference [9]. It has been reported that large-size spheres can be trapped more easily and delivered much faster than small ones [9,11]. Nevertheless, the observed propulsion speed of the trapped microparticles with diameters of 5 μm here is larger than that in the previous work using a slightly tapered telecom single-mode optical fiber and microparticles with diameters of 10 μm [13]. In addition to the contribution from the enhanced evanescent wave of the slightly tapered no-core optical fiber, we attribute this larger-speed phenomenon partly to the contribution from the fast flowing fluid. In the movie, we have found that the flow speed of an untrapped particle can reach as high as 154 μm·s^−1^ by using the particle video analysis. If the laser light source is replaced by a new one with an infrared wavelength [13], under which the optical absorption and heating effect of the silica glass fiber can be reduced, the observed propulsion speed will be at the same scale as the other reports [7,8,10,12,13]. The high propulsion speed of the trapped particle is also reported in Reference [11], where the wavelength of the used laser source is also 532 nm. On the other hand, the slight swinging of the temporal trace of the particle position along the tapered fiber results from the slightly nonuniform propulsion velocity, which is attributed to the residual polymer coating obstacles on the stripped tapered fiber surface. Because the slightly tapered no-core optical fiber is a multimode fiber with many guided modes and the spatial period of the slightly oscillating propulsion speed is approximately 150 μm, as indicated by Figure 4, we don't attribute it to the few-mode interference beating effect [14]. Yet, this issue about the propulsion speed variation still needs more detailed investigation by constructing a new experimental configuration and selecting a few-mode fiber [14].

The trapped microparticle continued to be propelled for a rather long range of ∼650 μm (see the movie Media 1 associated with Figure 3 from time 25 s to 59 s). Even in the part of the rear fiber taper with slightly lower scattered light intensity, we still could observe clearly the trapping and propelling of the microparticles. This illustrates a great advantage of using a slightly tapered no-core optical fiber to manipulate the microparticles, because the evanescent wave can always exist around the surface of a no-core optical fiber. However, a slightly tapered telecom single-mode optical fiber can have a strong enough evanescent wave to trap and propel microparticles only on the fiber surface nearby the tapered fiber waist [13]. Several other individual microparticles were also observed to be trapped and propelled along the surface of the slightly tapered no-core optical fiber. Due to the heating effect of the green laser light on the silica glass fiber and the resultant fluid convection, we could not demonstrate the case that the microparticles were propelled solely by the evanescent wave. Nevertheless, the stable evanescent wave trapping of the highly shining microparticles on the tapered fiber surface within fast flowing fluid was clearly observed in the movie.

As also shown in Figure 3, contrary to the other microparticles diffused in the water, the microparticle trapped on the fiber surface can emit strong light due to the coupling to the evanescent wave of the slightly tapered no-core optical fiber. The untrapped microparticles flowing near the surface of the slightly tapered no-core optical fiber at a distance ranging between 50 and 100 μm approximately can also emit some shining light as they encounter the leaking light, especially around the front half of the fiber taper. Nevertheless, due to the heating effect of the green laser light on the silica glass fiber and the resultant fluid convection, the flow speed of the microparticles is rather fast in the vicinity of the front half of the fiber taper, where the scattered light around the fiber surface is rather strong. Therefore several shining microparticles passing through the close neighborhood of the front half of the fiber taper were not trapped (Media 1). This drawback can be reduced if the laser light source is replaced by a new one with an infrared wavelength [13], under which the optical absorption and heating effect of the silica glass fiber can be reduced. Also, some microparticles were found to be stuck to the fiber surface due to effects such as the strong viscosity that results from the deterioration of a polystyrene microparticle [13]. Hence, as shown in Figure 3, some microparticles trapped on the fiber surface cannot move and have a zero propulsion velocity, although they can also emit strong light.

In the initial tests, we pre-stripped the polymer coating only from the section of the no-core optical fiber that would be given the tapering treatment. Yet, the measured fiber output power was only 4 mW when the 90 mW green laser light was launched into the no-core optical fiber. We attribute the abnormal high optical loss to the refractive index of the coating being too close to, or even larger than, that of the glass rod of the no-core optical fiber such that the light guiding is largely weakened. If we further stripped the coatings of both the untapered and unstripped sections of the no-core optical fiber after the previous tapering treatment, the tapered fiber could easily crack. Therefore, in a revised procedure we decided to first strip the coating from a much longer section of the no-core optical fiber. After the tapering treatment, the unstripped sections of the tapered fiber at the two far ends were then cut off. The remaining entirely stripped and slightly tapered no-core optical fiber (length ∼12 cm) was then used for the optical output test, and the measured fiber output power was finally raised to 45 mW for this case.

Since a stripped no-core optical fiber is itself a waveguide and the guided laser light can produce an evanescent wave on the fiber surface through total internal reflection, in the initial tests, we attempted to produce a tapered no-core optical fiber with a waist diameter tens of micrometers larger than 50 μm [13]. However, even though the fiber output power was rather strong, the scattered light around the tapered fiber surface did not appear to have high enough optical intensity and the resultant evanescent wave did not have large enough penetration depth or trapping force. Hence the loosely tapered no-core optical fiber was incapable of attracting the microparticles within fast flowing fluid in our tests. After we produced the tapered no-core optical fiber with a waist diameter ∼45 μm (slightly smaller than 50 μm [13]), which still far exceeds the subwavelength scale [7–12], we found that the scattered light around the tapered fiber surface was very strong, and we finally achieved trapping and propelling of microparticles by using this entirely stripped and slightly tapered no-core optical fiber. As a consequence, in our tests, even for a no-core optical fiber, it is necessary to perform the fiber tapering treatment in order to enhance the optical intensity of the evanescent wave and improve the attraction and delivery action on the microparticle around the fiber surface. However, as stated above, due to the heating effect of the green laser light on the silica glass fiber and the resultant fluid convection, the flow speed of the untrapped microparticles is rather fast in the vicinity of the front fiber taper. If the laser light source is replaced by a new one with an infrared wavelength [13], under which the optical absorption and heating effect of the silica glass fiber can be reduced, we believe that a tapered no-core optical fiber with a waist diameter tens of micrometers larger than 50 μm should also have an evanescent wave that is strong enough to trap and propel the microparticles.

The number of the transverse guided modes propagating in a fiber waveguide can be indicated approximately by the *V* parameter [12,14,16]:

(1) $$V=\frac{2\pi a}{\lambda }\sqrt{n_{\text{core}}^{2}-n_{\text{cladding}}^{2}}$$

where *λ* = 0.532 μm is the optical wavelength, *a* is the fiber radius, and *n*_core_ = *n*_silica_ = 1.46 and *n*_cladding_ = *n*_water_ = 1.33 are the core and cladding refractive indices, respectively. The number of guided modes can be further calculated approximately by [16]:

(2) $$M=\frac{4}{\pi^{2}}V^{2}$$

**Figure 5.**
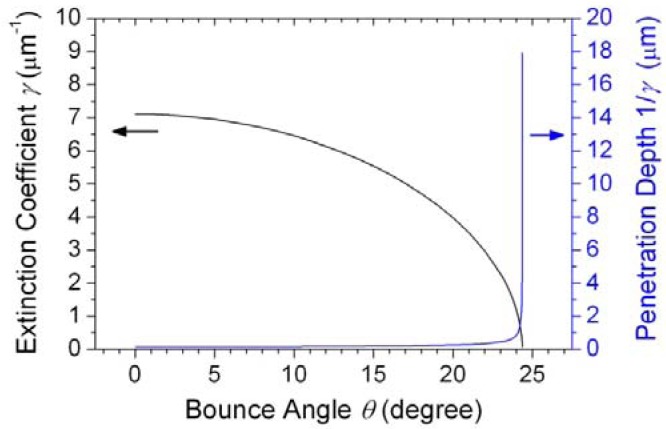
The calculated variations of the extinction coefficient *γ* and penetration depth 1/*γ* of the evanescent wave around the surface of a slightly tapered no-core optical fiber as a function of the bounce angle *θ* between the laser light propagation and the fiber-water interface by a planar-waveguide approximation.

**Figure 6.**
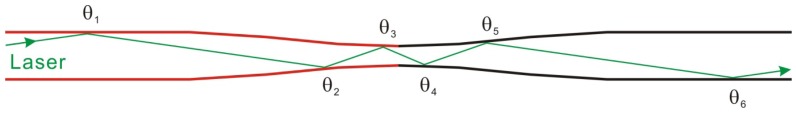
The illustration of the laser light propagation in a slightly tapered no-core optical fiber for various tapered fiber sections and bounce angles *θ*, which indicate implicitly various penetration depths, scattered light intensities, and trapping force magnitudes of the evanescent waves.

For an original no-core optical fiber of diameter 2*a* = 125 μm, we can have the parameter *V* = 445 and the mode number *M* = 80,095, and for a slightly tapered no-core optical fiber of waist diameter 2*a* = 45 μm, we can have the parameter *V* = 160 and the mode number *M* = 10,380. The number of the transverse modes propagating in a slightly tapered no-core optical fiber is indeed much larger than that in a heavily tapered optical fiber (subwavelength nanofiber) [7–12]. As a result, in a slightly tapered no-core optical fiber, the bounce angle between the higher-order-mode laser light propagation and the fiber-water interface will also be larger, producing an enhanced evanescent wave with bigger penetration depth and stronger trapping force as well. For an evanescent wave out of a no-core fiber waveguide with refractive index *n*_1_ = *n*_silica_ = 1.46, its electric field amplitude in the transverse *y*-direction in a surrounding water medium with refractive index *n*_2_ = *n*_water_ = 1.33 is proportional to exp(−*γ y*) by a planar-waveguide approximation. The extinction coefficient *γ* of an evanescent wave can be approximately expressed as [16]:

(3) $$\gamma =\frac{2\pi n_{2}}{\lambda }\sqrt{\frac{\cos^{2}\theta }{\cos^{2}{\bar{\theta }}_{\text{c}}}-1}$$

where *λ* = 0.532 μm is the optical wavelength, *θ* is the bounce angle between the laser light propagation and the fiber-water interface, and *θ̅_c_* = cos^−1^(n_2_/n_1_) = 24.4° is the complement of the total internal reflection critical angle. A plot of the extinction coefficient *γ* and penetration depth 1/*γ* of the evanescent wave *versus* the bounce angle *θ* is shown in Figure 5 under the angle range of 0 < *θ* < *θ̅_c_*. For higher-order modes in a slightly tapered no-core multimode optical fiber, when approaching the angle limit *θ̅_c_* (but not exceeding the angle limit *θ̅_c_*), the larger the bounce angle *θ*, the bigger is the penetration depth 1/*γ* of the evanescent wave, the stronger is the scattered light intensity, and the larger is the optical trapping force. As shown in Figure 3, when the laser light propagates from the untapered section to the front fiber taper, the scattered light around the fiber surface becomes stronger substantially. We attribute this enhanced evanescent wave to the bounce-angle difference (*θ*_1_ < *θ*_2_ < *θ*_3_) at the front sections of the tapered fiber, which is illustrated in Figure 6. Furthermore, as also shown in Figure 3, after passing through the tapered fiber waist, the scattered light around the surface of the rear fiber taper becomes weaker than that of the front fiber taper. We also attribute this phenomenon to the bounce-angle difference (*θ*_4_ > *θ*_5_ > *θ*_6_) at the rear sections of the tapered fiber, which is also illustrated in Figure 6. These events demonstrate again the advantage of the slightly tapered no-core multimode optical fiber over the slightly tapered telecom single-mode optical fiber when used in the evanescent wave trapping and propelling of microparticles. Besides, the above mechanism can also explain conceptually the aforementioned phenomenon that the loosely tapered no-core optical fiber was incapable of attracting the microparticles within fast flowing fluid in our tests. For a tapered no-core optical fiber with smaller waist diameter and bigger taper angle, the enhanced evanescent wave can have larger bounce angle, bigger penetration depth, and stronger trapping force.

## 4. Conclusions

We have achieved optical attraction and transportation of dielectric microparticles along the surface of an optical fiber within fast flowing fluid by using the enhanced evanescent wave from an entirely stripped and slightly tapered no-core optical fiber, to which the necessary laser light is easily coupled. The trapped microparticle continued to be propelled over a rather long delivery range. Like the slightly tapered telecom single-mode optical fiber, which also does not require a cumbersome heavy tapering treatment, a slightly tapered no-core multimode optical fiber has been shown to be another excellent and convenient tool for the optical propulsion of microparticles by the evanescent wave of a tapered fiber optical waveguide. Since a slightly tapered no-core optical fiber can be used to stably trap the microparticles in fast flowing fluid and propel at long range, revealing the strong trapping force due to the enhanced evanescent wave, this device has better potential than the alternative techniques that utilize a slightly tapered telecom single-mode optical fiber or a fragile subwavelength nanofiber for the application in manipulating large-size biological cells or microparticles in solution with tapered fiber sensors.

## Supplementary Files

Supplementary File 1 MOV-Document (MOV, 4052 KB)
